# CRISPR-Cas9-mediated targeted gene deletion in *Aspergillus calidoustus*, a non-model environmental mold

**DOI:** 10.1128/spectrum.03899-25

**Published:** 2026-05-11

**Authors:** Jeffrey M. Hollomon, Kurt M. Dahlstrom

**Affiliations:** 1 Department of Microbiology, Franklin College of Arts and Sciences, University of Georgia 1355, Athens, Georgia, USA; Universitat fur Bodenkultur Wien, Vienna, Austria

**Keywords:** *Aspergillus calidoustus*, CRISPR-Cas9, non-model organism, environmental isolate

## Abstract

Reverse genetic approaches in molds are complicated by their recalcitrance to transformation, multicellular growth during much of the vegetative life cycle, and frequent off-target integration of deletion constructs. Thus, protocols for gene deletion in filamentous fungi are predominantly confined to established model organisms. Adapting an existing protocol developed for gene deletion in the related fungus *Aspergillus fumigatus*, we employed an expression-free CRISPR/Cas9 directed mutagenesis strategy to the non-model environmental mold *Aspergillus calidoustus*. As a test case, we have deleted *A. calidoustus pyrG*, encoding orotidine-5′-phosphate decarboxylase, using short regions of homology to guide on-target integration of a nourseothricin resistance cassette (NatR) to CRISPR/Cas9-induced double strand breaks. We genotypically and phenotypically validated two *A. calidoustus* Δ*pyrG* deletion strains generated using this methodology, with whole-genome sequencing revealing one Δ*pyrG* strain to have integrated the resistance cassette by homologous recombination, and another strain by non-homologous end joining. Thus, distinct modes of double-strand break repair were responsible for on-target integration of the homology-bearing NatR cassette at the *pyrG* locus in *A. calidoustus*.

**IMPORTANCE:**

In the environment, filamentous fungi play essential roles in soil health and agriculture, decomposition, and nutrient cycling. The study of these organisms is often limited by an inability to dissect the functions of their genes and the roles that they play by modifying the genomes of these organisms. Here, we adapt and validate a tool for genome modification to *Aspergillus calidoustus*, a soil fungus that forms a partnership with a bacterium (*Paraburkholderia edwinii*) to resist natural toxins found in the soil.

## INTRODUCTION

Owing to their importance in infectious disease, plant pathogenesis, biotechnology, and basic research in molecular biology, *Aspergillus* spp. have long been a focus of genetic research. Reverse genetic approaches in these fungi have traditionally been frustrated by low efficiencies of extant transformation methodologies and a preference in these organisms favoring non-homologous end joining (NHEJ) over homology-directed repair, which is typically required for insertion of a selectable marker at a defined locus of interest (1). CRISPR-Cas9-based approaches have, to an extent, ameliorated these challenges in the genetic manipulation of molds. CRISPR-Cas9 genome editing in filamentous fungi initially required genomic integration of the Cas9 machinery or expression of Cas9 and gRNAs from extrachromosomal plasmids; recently, genome editing by transformation with Cas9 ribonucleoproteins has been demonstrated (2–7). Additionally, it has been found that the induction of CRISPR-Cas9 double-strand breaks permits the use of short regions of homology to target disruption cassettes to a locus of interest, obviating the past requirement to append large regions of homology to resistance markers (8). In the human pathogenic fungus *Aspergillus fumigatus*, Al Abdallah et al. combined this approach of using short regions of target homology with expression-free CRISPR-Cas9 genome editing by preassembled ribonucleoproteins (9).

Working in non-model, environmental *Aspergilli* complicates reverse genetic strategies further than the challenges addressed above, as these organisms possess intact NHEJ machinery, lack auxotrophies, and do not benefit from well-established transformation protocols. *Aspergillus calidoustus* ADI1 is a soil-derived fungus that was isolated in association with a partner bacterium, *Paraburkholderia edwinii*. When cultured together, *P. edwinii* protects *A. calidoustus* from a number of antifungal compounds that otherwise would inhibit its growth (10). Notable among these is phenazine-1-carboxylic acid, a redox-active heterotricyclic metabolite commonly found in soil and produced by *Pseudomonas* spp. (reviewed in reference 11). To enable future exploration through reverse genetics of the mechanistic basis of this interkingdom protection phenomenon, we have adapted the expression-free approach previously employed in *A. fumigatus* to the environmental filamentous fungus *A. calidoustus*, which we report here. This approach, which employs transformation of protoplasts (a single-celled propagule upon which selection can be performed without subsequent sporulation), requires no cloning to generate locus-specific deletion cassettes, and the transformation can be carried out by a single researcher in a single day. The gene encoding orotidine-5′-phosphate decarboxylase (*pyrG*) was selected as an initial target for directed mutagenesis in *A. calidoustus* due to its thorough characterization in other species, with well-defined selectable phenotypes for both its presence and absence.

## MATERIALS AND METHODS

### Strains and growth conditions

All strains used in this study can be found in Table S1. Experiments were conducted on potato dextrose agar and broth, the media on which the *A. calidoustus* was initially isolated, as attempts to culture *A. calidoustus* in a minimal medium (a modified *Aspergillus* glucose minimal medium containing the following trace metals in lieu of Hutner’s trace elements: 1 μM Fe, 0.0797 μM Zn, 0.121 μM Mn, 0.05 μM Co, 0.1 μM Mo, 0.0196 μM Cu, 0.01 μM Se, and 0.19 μM Ni) did not produce robust growth (12). Selection of transformants was carried out on potato dextrose agar, osmotically stabilized with 1.2 M sorbitol, and supplemented with 200 μg/mL nourseothricin and/or 10 mM uracil and 5 mM uridine where appropriate.

### Plasmid construction and oligonucleotide reagents

Nourseothricin acetyltransferase from *Streptomyces nourseii* (Uniprot Q08414) was synthesized as double-stranded DNA and cloned by Gibson assembly into pCSN44, replacing the hygromycin resistance marker. This yielded pJMH8, which contains an ORF encoding nourseothricin acetyltransferase (NatR) under the control of the *A. nidulans trpC* promoter and terminator. Primers and crRNA sequences used in this study are found in Table S2.

### Generation, isolation, and transformation of *Aspergillus calidoustus* protoplasts

Mycelial tissue grown from spores (conidia) in potato dextrose broth (PDB)/.1% Tween-20 for 48 h at 30°C with 250 RPM shaking were harvested via Büchner funnel, washed three to four times with sterile phosphate-buffered saline, and rinsed with osmotic medium (1.2 M MgSO_4_, 10 mM sodium phosphate, pH 5.8). Six grams of tissue were divided into two 50 mL conical tubes, resuspended in 35 mL osmotic medium each, and 500 mg of Vinotaste Pro (Novonesis) was added, followed by brief vortexing. These digestions were then incubated at 30°C with gentle agitation (80 RPM on a shaking platform) for 4 h, with brief, gentle vortexing every hour. Following digestion, digestions were carefully overlaid with 5 mL trapping buffer (0.6 M sorbitol, 100 mM Tris, pH 7.5) and centrifuged at 3,800 RCF at 4°C for 20 min with an intermediate deceleration setting in a swinging bucket rotor. Protoplasts appeared as a hazy layer at the interface of the trapping buffer and osmotic medium. Five milliliters of the interface, containing protoplasts, from each digestion were pipetted into a new conical tube, and 40 mL of ice-cold STC buffer (1 M sorbitol, 10 mM CaCl_2_, 10 mM Tris, pH 7.5) was added, followed by gentle mixing through inversion. Protoplasts were then pelleted by centrifugation at 3,550 RCF at 4°C for 20 min, supernatant decanted, and protoplasts resuspended in 1 mL ice-cold STC and transferred to a microcentrifuge tube. Protoplasts were pelleted and washed once in 1 mL ice-cold STC by centrifugation for 3 min at 2,400 RCF in a pre-chilled rotor. Supernatant was gently aspirated, and protoplasts resuspended in a volume of ice-cold STC corresponding to the number of transformations being carried out (70 μL for each transformation, and 30 μL for each no-DNA negative control).

crRNAs were designed using IDT Custom Alt-R CRISPR-Cas9 guide RNA design tool, and commercially synthesized by IDT. Cas9 ribonucleoproteins (RNPs) were assembled by incubating 1.5 μg Alt-R S.p. Cas9 Nuclease V3 (a recombinant Cas9 containing the SV40 nuclear localization sequence, IDT cat. number 1081058) with 50 picomoles of each sgRNA duplex (targeting the 5′ and 3′ untranslated regions of *pyrG*, and consisting of equimolar crRNA and Alt-R CRISPR-Cas9 tracrRNA (IDT cat. number 1072533)) in a Cas9 working buffer (150 mM KCl, 20 mM HEPES, pH 7.5). To transform protoplasts, 400 ng of repair construct DNA (osmotically stabilized in two volumes STC buffer), and Cas9 ribonucleoproteins (1.5 μg of Cas9 loaded with 50 picomoles of each crRNA/tracrRNA duplex, osmotically stabilized in one volume STC buffer), were added to 200 μL PEG solution (60% wt/vol polyethylene glycol-6000, 10 mM CaCl_2_, 10 mM Tris, pH 7.5), followed by 70 μL resuspended protoplasts. This mixture was incubated on ice for 30 min. One milliliter of PEG solution was then added, mixed thoroughly by pipetting, and incubated on ice for a further 15 min.

To plate transformants, 20 mL of osmotically stabilized PDA (1.2 M sorbitol, .5% agar), supplemented with 10 mM uracil, 5 mM uridine, and 200 μg/mL nourseothricin, was added to the transformation mixture, mixed thoroughly by gentle inversion, and decanted over two plates of PDA (1.2 M sorbitol, 1.75% agar, 10 mM uracil, 5 mM uridine, and 200 μg/mL nourseothricin). Plates were incubated at 30°C, with visible colonies appearing between 48 and 72 h.

### Genotypic characterization of candidate *ΔpyrG* mutants by PCR

DNA was extracted from plate-grown mycelia by bead beating (6 m/s for two 1-min intervals, with a 5-min stationary rest between intervals), followed by potassium acetate precipitation of protein, a chloroform extraction, and isopropanol precipitation of DNA. PCR was carried out using primers flanking the *pyrG* locus to assess disruption of the wild-type (WT) allele and integration of the NatR resistance cassette.

### Genome sequencing

Mycelial tissue was grown from spores in potato dextrose broth supplemented with 10 mM uracil and 5 mM uridine, and grown for 48 h with 250 RPM shaking at 30°C. Mycelia were harvested by Büchner funnel under vacuum, and washed three to four times with phosphate-buffered saline, and ground under liquid nitrogen in a mortar and pestle. Genomic DNA was purified from pulverized mycelia using the Wizard HMW DNA Extraction Kit (Promega cat. number A2920). Genomic DNA was sequenced at Plasmidsaurus using Oxford Nanopore sequencing. Genome assemblies were generated by first removing the bottom 5% worst fastq reads via Filtlong v0.2.1 (with heavy weight applied to remove low-quality reads, --mean_q_weight 10), and running hifiasm version 0.25.0 with parameters for high-quality ONT reads. Genome completeness was assessed with BUSCO version 5.8.0.

## RESULTS

### *Aspergillus calidoustus* exhibits sensitivity to nourseothricin

Nourseothricin, a broad-spectrum polyketide protein synthesis inhibitor, is a dominant selection marker that has been employed for selection in *Aspergillus niger* and *Aspergillus nidulans* (13, 14). To determine the suitability of the dominant resistance marker nourseothricin acetyltransferase (NatR), which confers resistance to nourseothricin, wild-type *A. calidoustus* spores (conidia) were plated on PDA containing nourseothricin at a concentration of 200 μg per mL. *A. calidoustus* displayed sensitivity to nourseothricin at this concentration, with visible growth fully inhibited at 48 h.

### PEG-mediated transformation of *A. calidoustus* protoplasts yields nourseothricin-resistant colonies

**Fig 1:**
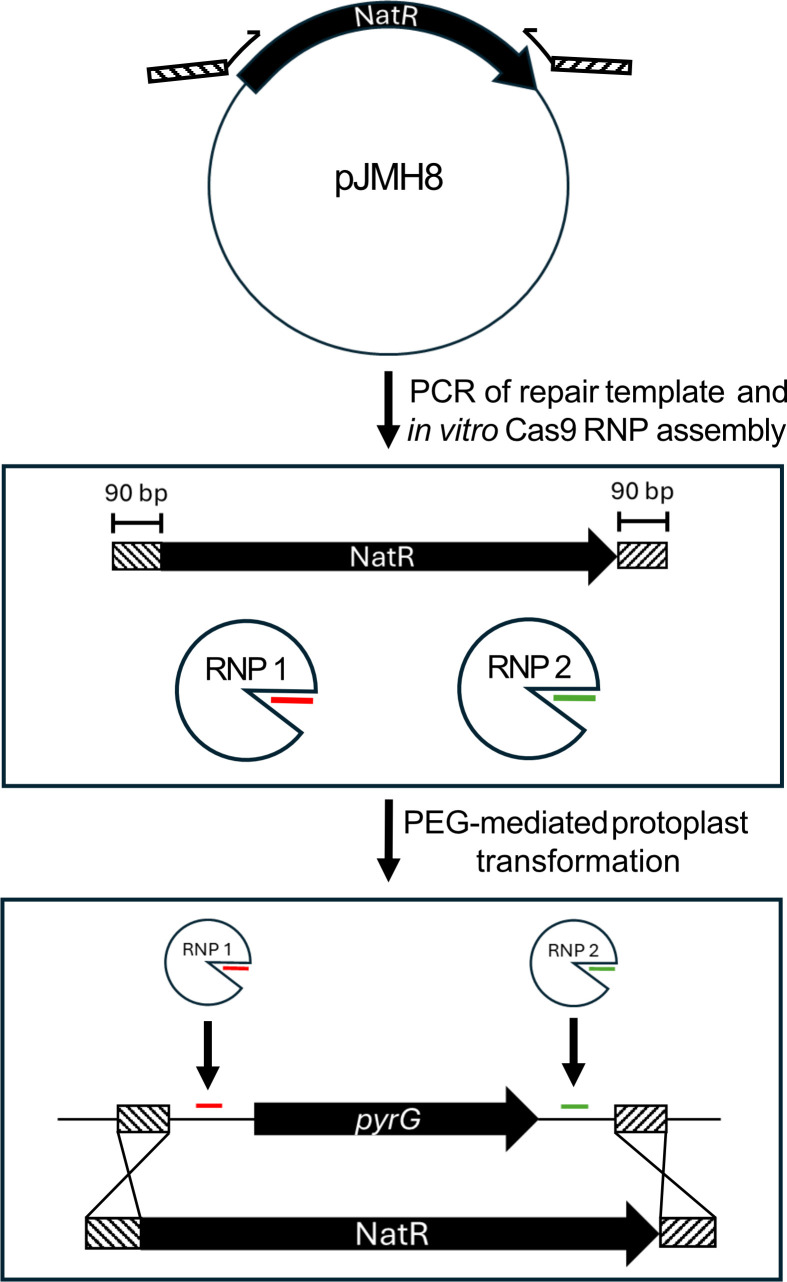
Schematic overview of mutagenesis strategy. Ninety base pairs of target-specific homology to the *Aspergillus calidoustus pyrG* locus was appended to a nourseothricin resistance (NatR) cassette through integration into PCR primers. Cas9 ribonucleoproteins (RNPs) targeting the 5′ and 3′ untranslated regions were assembled *in vitro* and transformed alongside the site-specific NatR cassette by polyethylene glycol-mediated transformation of enzymatically derived protoplasts. Sites targeted by Cas9 are marked in red and green, and regions of target-specific homology are marked with hatching.

A putative *pyrG* homolog, encoding orotidine-5′-phosphate decarboxylase, was identified in the genome of *A. calidoustus* by translated BLAST of the *Aspergillus nidulans* PyrG protein sequence against the *A. calidoustus* genome. crRNAs targeting both the 5′ and 3′ untranslated regions of *pyrG* were loaded into commercially acquired recombinant Cas9 nuclease and transformed with PEG into protoplasts alongside a repair construct containing a nourseothricin resistance marker with 90 base pairs of flanking homology to the *pyrG* locus (Fig. 1). Homology was appended to the resistance marker by integration into PCR primers and amplification of the marker from pJMH8, and this homology flanked the CRISPR-Cas9 cut sites to ensure Cas9 would not cut the repair construct. Transformed protoplasts were subsequently plated onto medium containing 200 μg/mL nourseothricin, and incubated at 30°C. Between 48 and 72 h, resistant colonies became visible, and were counted. Using 400 ng of repair construct DNA targeting the *pyrG* locus yielded an average of 46 transformants per transformation (with a standard deviation of 19), from which we extrapolate a transformation efficiency of 115 transformants per microgram of repair construct DNA. These values represent the average of three independent transformations carried out over three separate days.

### CRISPR-Cas9 targeting of the *pyrG* locus

**Fig 2:**
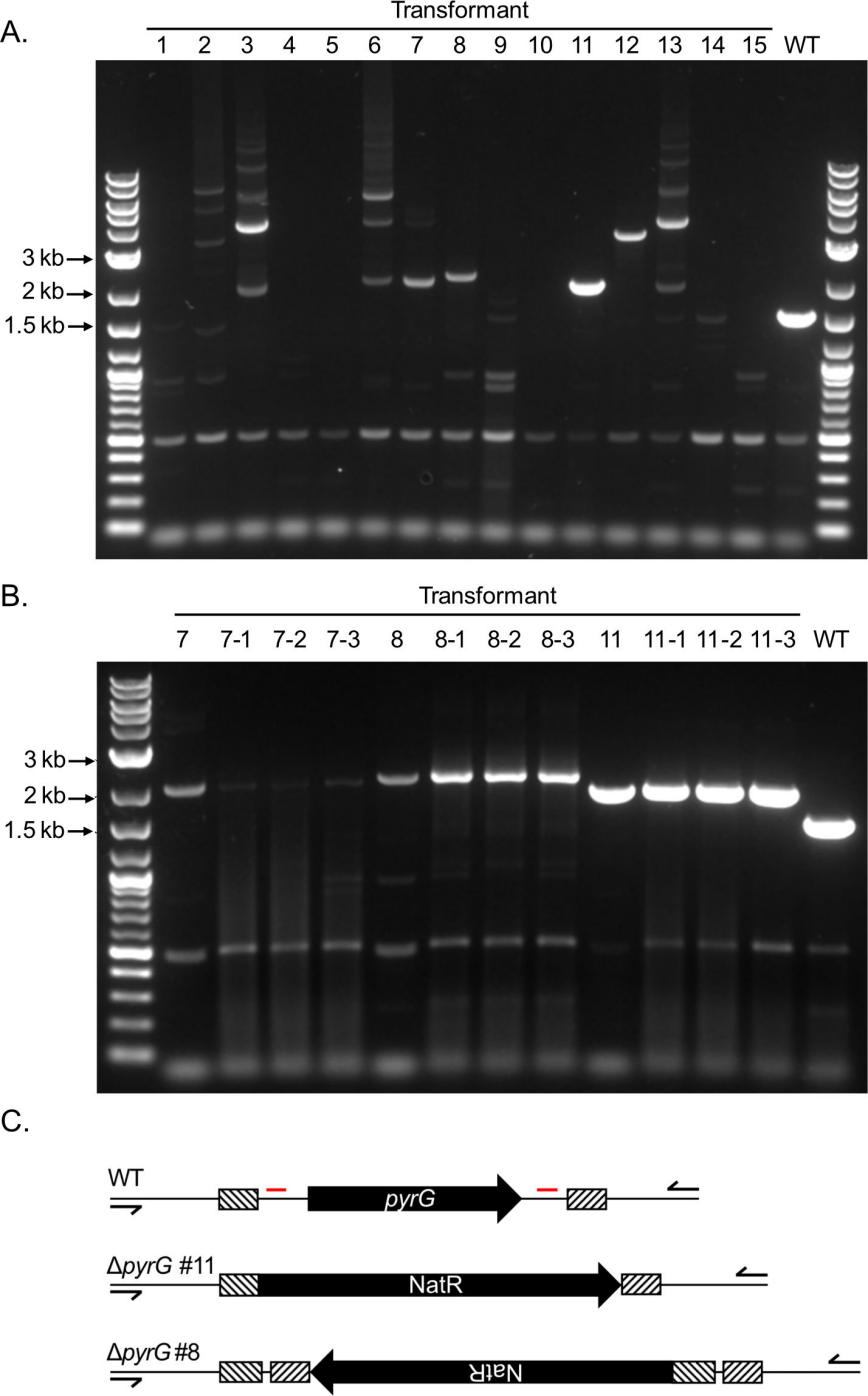
Disruption of the *pyrG* locus assessed by PCR. (**A**) PCR amplification of genomic DNA of first-round transformants with primers flanking the *pyrG* locus. The undisrupted locus has a length of 1,518 bp, whereas the correct integration of the NatR cassette by homologous recombination has a predicted length of 1,976 bp. The terminal lanes contain the 1 kb Plus DNA Ladder (NEB). (**B**) PCR amplification of the *pyrG* locus in three clones from transformants 7, 8, and 11 following single-spore purification. Lanes marked 7, 8, and 11 are the PCR reactions seen in panel A, and rerun alongside their progeny for reference. (**C**) Schematic representation of the *pyrG* locus in WT ADI1 and Δ*pyrG* transformants 8 and 11. sgRNA targets of Cas9 are marked in red, and regions of homology integrated into the NatR resistance cassette marked with hatching. Location of primers used in panels A and B are indicated.

Fifteen transformants were excised and transferred to PDA plates containing nourseothricin and allowed to grow radially. Mycelial tissue was excised from the growing colonies, and its genomic DNA was extracted. To assess on-target integration of the homology-directed repair cassette into the *PYRG* locus, primers were designed to flank the region of homology-directed repair, and the locus amplified from genomic DNA isolated from the transformants and a wild-type control (Fig. 2A). While all transformants except 1, 9, 13, and 14 showed the absence of an amplicon corresponding to the wild-type *pyrG* allele, only three (7, 8, and 11) possessed bands that approximately corresponded to correct integration of a single NatR repair construct. To ensure these transformant populations putatively integrating the NatR marker into the *pyrG* locus were isogenic, and not the genetically mixed progeny of a heterokaryotic transformant, spores were collected from three transformants (7, 8, and 11), plated for isolation, and single colonies were excised and transferred to PDA plates containing nourseothricin. Following the growth of three independent isolates from each candidate mutant, mycelial tissue was again collected, genomic DNA extracted from it, and the *pyrG* locus interrogated by PCR. In the progeny of transformants 8 and 11, we observed bands corresponding to their parental strain (Fig. 2B). To further increase the likelihood that these transformants were truly isogenic, we again isolated to single spores, for a total of two single-spore isolations following initial transformant selection.

### Whole-genome sequencing revealed two distinct patterns of integration at the *pyrG* locus

To assess the integration of the resistance marker at the *pyrG* locus, and to investigate the possibility of ectopic integration of the marker at off-target loci, we sequenced the genomes of transformants 8 and 11 after the two rounds of single spore purification described above (summary statistics in Table S3). Consistent with our findings by PCR, both strains integrated a single copy of the NatR marker at the *pyrG* locus, with the wild-type allele deleted between the CRISPR cut sites in the 5′ and 3′ UTRs of the gene (Fig. 2C; File S1). From the orientation and insertion point of the resistance cassette, we concluded transformant 11 integrated the resistance cassette in a manner consistent with homologous recombination at the flanks of the marker containing homology to the *pyrG* locus. Transformant 8, however, integrated the cassette in the reverse orientation, and retained the homology on each marker flank from the opposite UTR, consistent with integration via non-homologous end joining at both CRISPR cut sites. This extra sequence accounts for the observation that the PCR product amplified from transformant 8 ran slightly larger than predicted, as shown in Fig. 1. While expression-free CRISPR-Cas9 mutagenesis through transformation with ribonucleoproteins has been described to be highly specific and possessing a low potential for off-target integration in the related fungus *A. fumigatus*, we sought to rule out the possibility of ectopic integration of the resistance cassette in our Δ*pyrG* strains (15). To subject the genomes to an unbiased query for ectopic integration of the resistance cassette, BLAST databases were generated from the genomes of both mutant strains, and we were unable to identify any loci of ectopic integration of the marker in either Δ*pyrG* mutant strain by BLAST of the resistance marker against their genomes.

### *Aspergillus calidoustus* Δ*pyrG* obligately requires uracil and uridine supplementation and is resistant to 5-FOA

**Fig 3:**
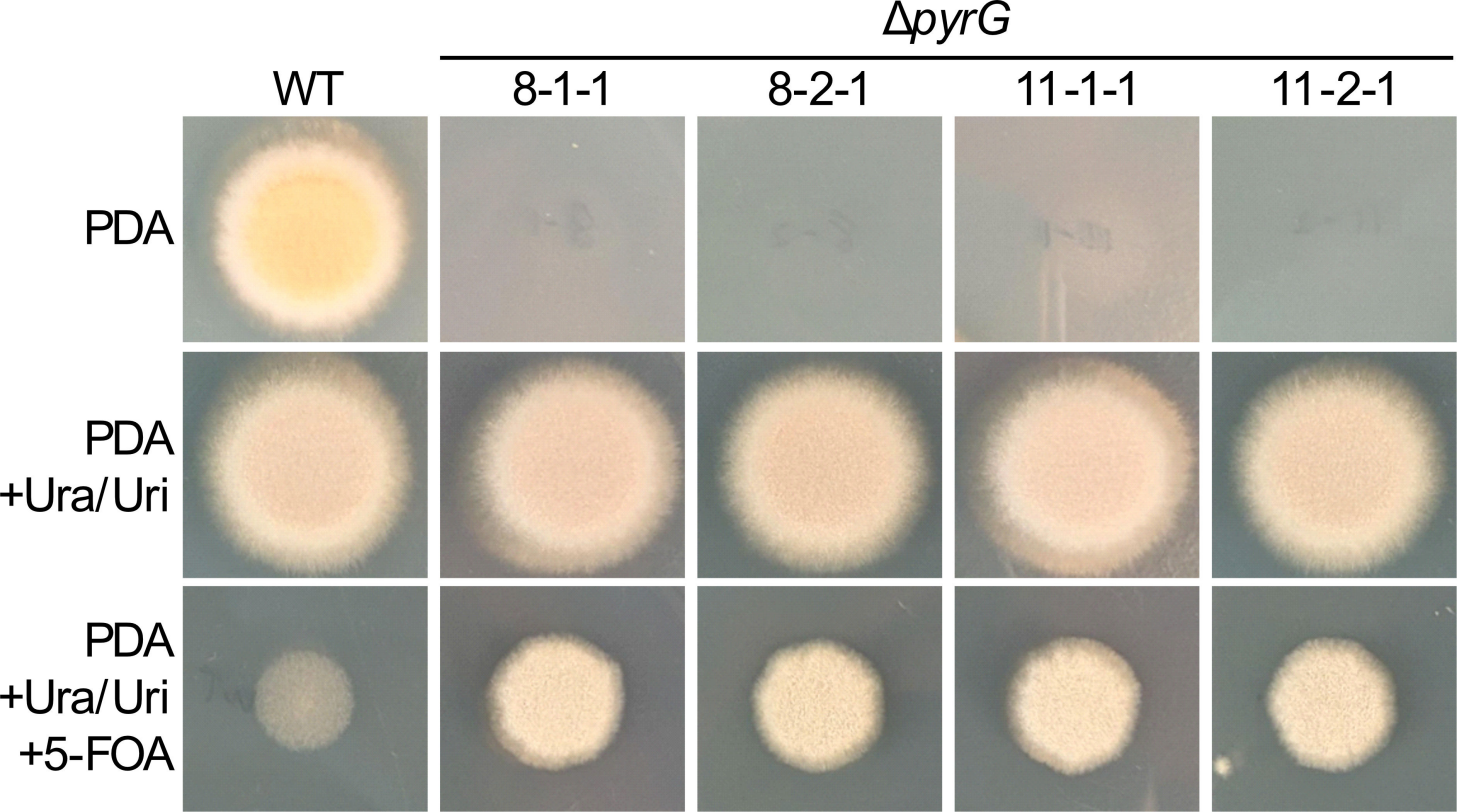
*Aspergillus calidoustus* Δ*pyrG* exhibits a pyrimidine requirement and 5-FOA resistance. 10^5^ wild-type or Δ*pyrG* spores were spotted onto PDA without supplement, PDA containing 10 mM uracil and 5 mM uridine, and PDA containing 10 mM uracil, 5 mM uridine, and 1 mg/mL 5-FOA. Plates were incubated at 30°C with growth observed at 48 h. Two clones from transformants 8 and 11 were assayed following two rounds of single spore purification.

As the *A. calidoustus* genome encodes a single copy of *pyrG*, disruption of *pyrG* should result in an obligate auxotrophy for the pyrimidines uracil and uridine, and confer resistance to 5-fluoroorotic acid (5-FOA) by preventing the conversion by the PyrG enzyme of that prodrug to the highly cytotoxic 5-fluorouracil. To ascertain the phenotypic consequences of *pyrG* disruption on *A. calidoustus*, spores from both Δ*pyrG* mutant strains, alongside wild type, were plated on solid medium with and without pyrimidine supplementation (10 mM uracil and 5 mM uridine), as well as with pyrimidine supplementation and 5-FOA. Consistent with loss of *pyrG* function, both mutant strains lost the ability to grow in the absence of exogenous uracil and uridine but gained the ability to grow on medium containing 5-FOA (Fig. 3). By comparison, the growth of the WT was substantially diminished in the presence of 5-FOA. Taken together, these data are consistent with *pyrG* disruption in transformants 8 and 11.

### Cas9 targeting of the *pyrG* locus increases transformation efficiency

To determine the contribution of Cas9 targeting to the efficiency of transformation, we conducted a set of transformations with and without Cas9 RNPs. In these experiments, transformation with both RNPs and the NatR repair construct yielded a similar transformation efficiency to the previous set of transformations (117 transformants per µg of DNA, extrapolated from an average of 46.7 transformants per 400 ng transformation) (Table 1). Across three independent replicate transformations, Cas9 targeting significantly increased the average number of transformants compared to a control transformation with the DNA repair construct but lacking RNPs (46.7 vs 5, *P* = 0.013). As expected, transformation with RNPs, but without the repair construct, and thus, without the resistance marker to the drug selection, yielded very few to no transformants, as did a negative control transformation with neither DNA nor RNPs. Additionally, we determined, as a function of protoplast number, the efficiency of transformation with Cas9 RNPs and 400 ng of the DNA repair construct to be an average of 10.2 transformants per 10^5^ protoplasts (with a standard deviation of 4.9 per 10^5^ protoplasts), but this value is likely to be an overestimate due to the challenges of counting protoplasts on a hemocytometer (their propensity to clump together and to lyse under handling).

**TABLE 1:** Transformant yields from transformations with and without Cas9 ribonucleoproteins and DNA repair constructs targeting the *pyrG* locus

	Replicate 1	Replicate 2	Replicate 3	Average	Standard deviation
DNA and RNPs	63	42	35	46.7	11.9
DNA only	8	4	3	5.0	2.2
RNPs only	2	0	0	0.7	0.9
No DNA, No RNPs	1	0	0	0.3	0.5

**Fig 4:**
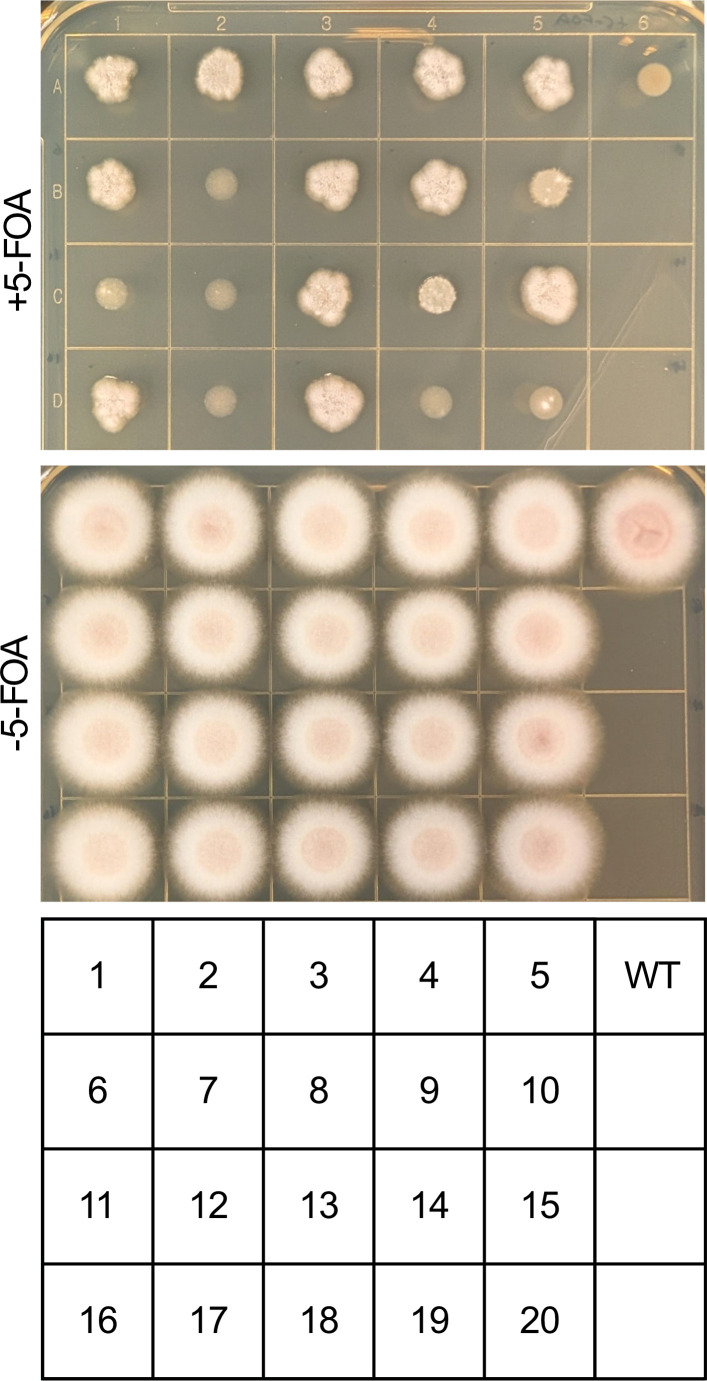
Screening of Cas9 RNP and NatR repair construct transformants for 5-FOA resistance. Spore suspensions in PBS of wild-type *A. calidoustus* and 20 transformants were spotted onto PDA/5 mM uridine/10 mM uracil with and without 1 mg/mL 5-FOA, incubated at 30°C, and observed at 72 h. As these transformants were generated separately from those in previous figures, the strain numbering is distinct and does not correspond to any strains described in previous figures.

Having generated an additional, independent set of transformants through experiments testing the role of Cas9 RNPs and repair DNA in transformation efficiency, we employed these to confirm the capacity of this methodology to disrupt *A. calidoustus pyrG* through phenotypic validation. To that end, we isolated twenty transformants that had been transformed with both Cas9 RNPs and the *pyrG* repair construct and screened these for resistance to 5-FOA (Fig. 4). Twelve of the 20 transformants exhibited robust growth on medium containing 5-FOA, consistent with *pyrG* loss-of-function in that subset of transformants.

## DISCUSSION

In this report, we demonstrate the suitability of an expression-free CRISPR/Cas9 mutagenesis method for targeted mutagenesis in the non-model filamentous fungus *Aspergillus calidoustus*.

CRISPR-Cas9 nuclease targeting combined with short regions of homology and nourseothricin selection was highly effective in disrupting the *pyrG* locus, with almost all transformants bearing PCR profiles consistent with some level of disruption. Of 15 transformants, we selected 2 for thorough genotypic and phenotypic characterization. While both bore the phenotypic stigmata of *pyrG* disruption, whole-genome sequencing revealed mutants of distinct natures, with one generated by homologous recombination and the other through non-homologous end joining. DNA repair through non-homologous end joining provides a mechanism for concatemeric insertion of repair constructs, and consistent with non-homologous end joining being a major contributor to double-strand break repair during transformation of *A. calidoustus*, several of the transformants (3, 6, and 13) displayed PCR profiles of the *pyrG* locus consistent with mixed populations bearing concatemeric insertion of the resistance marker (several high molecular weight bands corresponding to multiples of the molecular weight of the resistance marker). The presence of multiple bands in PCR reactions from these strains could be the result of concatemerization generating repeat sequences at the *pyrG* locus permitting expansion and collapses in PCR, generating multiple products.

We went on to determine the impact of Cas9 ribonucleoproteins on transformation efficiency. Cas9 RNPs increased transformation efficiency with the NatR repair construct by almost 10-fold, suggesting that the induction of double-strand breaks substantially increases the likelihood of integration of the repair construct into the *A. calidoustus* genome, even in an ectopic fashion. We then phenotypically screened a subset of this independent set of transformants for 5-FOA resistance, finding over half of the strains resulting from transformation with both Cas9 RNPs and the NatR repair construct to grow in the presence of 5-FOA, consistent with *pyrG* disruption.

We are currently exploring several avenues to increase efficiency of single integration at target loci. In *A. fumigatus*, *ku80* (required for double-strand break repair by non-homogolous end joining) has been found to be dispensable for CRISPR-Cas9 mutagenesis utilizing short regions of homology, so to reduce the occurrence of non-homologous end joining and promote mutagenesis through homologous recombination in *A. calidoustus*, and thus diminishing the incidence of concatenated insertion, work is ongoing in our group to generate a strain deficient in *ku80* (8). Although background was limited using nourseothricin as a selection marker (Table 1, one to two colonies appearing in transformations without the NatR resistance cassette in a single replicate), it can become more prevalent beyond 72 h of selection, which complicates the generation of mutants which might have a growth defect. To address this, and to allow for the deletion of multiple genes, as well as the complementation of deletion mutants, we are working to refine and expand strategies for selection. To that end, we are exploring the use of a hygromycin resistance marker, as well as employing the Δ*pyrG* strains developed in this study as a background, in which selection can be carried out through the restoration of pyrimidine prototrophy with the *pyrG* gene, and potentially recycled out through 5-FOA selection.

## Data Availability

Whole-genome sequences generated in this study have been deposited in the NCBI Biosample database under accession numbers SAMN54525906 and SAMN54525907.

## SUPPLEMENTAL MATERIAL

Data S1 spectrum.03899-25-s0001.txt Annotated pyrG locus nucleotide sequences of wild-type and mutant strains.

Table S1 spectrum.03899-25-s0002.docx Strains and plasmids used in this study.

Table S2 spectrum.03899-25-s0003.docx Oligonucleotide reagents used in this study.

Table S3 spectrum.03899-25-s0004.docx Genome assembly summary statistics.

ASM does not own the copyrights to Supplemental Material that may be linked to, or accessed through, an article. The authors have granted ASM a non-exclusive, world-wide license to publish the Supplemental Material files. Please contact the corresponding author directly for reuse.
