# Supplementary material for: CRISPR-Cas9-mediated targeted gene deletion in Aspergillus calidoustus, a non-model environmental mold
Source: Microbiol Spectr. 2026 May 11;14(6):e03899-25. doi: 10.1128/spectrum.03899-25 (PMC13228013; doi:10.1128/spectrum.03899-25)
Supplement: Table S1 — Strains and plasmids used in this study. [file spectrum.03899-25-s0002.docx]

Table S1: Strains and plasmids used in this study.

| Strain: | Genotype: | Parental: | Description: | Reference: |
| --- | --- | --- | --- | --- |
| *Aspergillus calidoustus* ADI1 | Wild-type | N/A | Soil isolate parental of mutants generated in this study. | Dahlstrom and Newman, 2022. |
| *Aspergillus calidoustus ΔpyrG* #8 | *pyrG::NatR* | *Aspergillus calidoustus* ADI-01 |  | This study. |
| *Aspergillus calidoustus ΔpyrG* #11 | *pyrG::NatR* | *Aspergillus calidoustus* ADI-01 |  | This study. |
|  |  |  |  |  |
| Plasmid: |  |  |  |  |
| pCSN44 |  | pBSSK+ | Contains a hygromycin resistance marker under the *A. nidulans trpC* promotor and terminator | Staben, et al. 1989. |
| pJMH8 |  | pCSN44 | Contains a nourseothricin resistance marker under the *A. nidulans trpC* promotor and terminator | This study. |
