# Supplementary material for: CRISPR-Cas9-mediated targeted gene deletion in Aspergillus calidoustus, a non-model environmental mold
Source: Microbiol Spectr. 2026 May 11;14(6):e03899-25. doi: 10.1128/spectrum.03899-25 (PMC13228013; doi:10.1128/spectrum.03899-25)
Supplement: Table S2 — Oligonucleotide reagents used in this study. [file spectrum.03899-25-s0003.docx]

Table S2: Oligonucleotide reagents used in this study. Gene-specific elements of deletion primers and crRNAs are underlined.

| 5' crRNA sequence | GGACGGAGCAAAAAUUCCACGUUUUAGAGCUAUGCU |
| --- | --- |
| 3' crRNA sequence | GCAUUUAGAGGUUGCAACUUGUUUUAGAGCUAUGCU |
| 5' deletion primer | GCAATTGCATGAATTTGACACGCCATTGTTTTGATTGAGGTCTTGGCAAAATTAAGTGCTTATCTTACTTATCGGCGCAGATGCAGATCGCAGAAGATGATATTGAAGGAGCAC |
| 3' deletion primer | CAAGCTATTCAAGTCTATACTCGTCCAGTGCGTTGGCAGGGTTGAGTCTGGTTGATAGGTTGCGTAATGTAGGTCTTTTATAGGCATGTATCTAGAAAGAAGGATTACCTCTAAACAA |
| 5' confirmation primer | ACACGGGATGACTTCTGGAATC |
| 3' confirmation primer | AGGTGTCAAGTTGTGGAGACC |
