# Supplementary material for: CRISPR-Cas9-mediated targeted gene deletion in Aspergillus calidoustus, a non-model environmental mold
Source: Microbiol Spectr. 2026 May 11;14(6):e03899-25. doi: 10.1128/spectrum.03899-25 (PMC13228013; doi:10.1128/spectrum.03899-25)
Supplement: Table S3 — Genome assembly summary statistics. [file spectrum.03899-25-s0004.docx]

Table S3: Summary statistics of Δ*pyrG* genome sequencing and assembly.

|  | **Δ*pyrG* transformant 8** | **Δ*pyrG* transformant 11** |
| --- | --- | --- |
| Genome Size: | 41.8 Mb | 40.5 Mb |
| Base pairs sequenced: | 1,769,997,578 bp | 1,474,093,009 bp |
| Total number of reads: | 185,966 | 147,869 |
| Longest read: | 120,904 bp | 123,453 bp |
| Estimated Coverage: | 42x | 36x |
| Contigs: | 54 | 33 |
| N50: | 4,685,793 bp | 4,685,578 bp |
| **BUSCO statistics** |  |  |
| Complete percentage | 98.6 | 98.6 |
| Complete BUSCOs | 4132 | 4132 |
| Multicopy BUSCOs | 11 | 11 |
| Fragmented Buscos | 4 | 4 |
| Missing BUSCOs | 55 | 55 |
| n_markers | 4191 | 4191 |
| BUSCO lineage | eurotiales_odb10 | eurotiales_odb10 |
